# Defining the optimal bilirubin level before hepatectomy for hilar cholangiocarcinoma

**DOI:** 10.1186/s12885-020-07385-0

**Published:** 2020-09-23

**Authors:** Wong Hoi She, Tan To Cheung, Ka Wing Ma, Simon H. Y. Tsang, Wing Chiu Dai, Albert C. Y. Chan, Chung Mau Lo

**Affiliations:** Department of Surgery, The University of Hong Kong, 102 Pok Fu Lam Road, Hong Kong, China

**Keywords:** Bilirubin level, ERCP, Hepatectomy, Hilar cholangiocarcinoma, Preoperative biliary drainage, PTBD

## Abstract

**Background:**

In the management of operable hilar cholangiocarcinoma (HC) patients with hyperbilirubinemia, preoperative biliary drainage is a measure to bring down the bilirubin to a certain level so as to avoid adverse postoperative outcomes that would otherwise result from hyperbilirubinemia. A cutoff value of bilirubin level in this context is needed but has not been agreed upon without controversy. This retrospective study aimed to identify a cutoff of preoperative bilirubin level that would minimize postoperative morbidity and mortality.

**Methods:**

Data of patients having hepatectomy with curative intent for HC were analyzed. Discriminative analysis was performed to identify the preoperative bilirubin level that would make a survival difference. The identified level was used as the cutoff to divide patients into two groups. The groups were compared.

**Results:**

Ninety patients received hepatectomy with curative intent for HC. Their median preoperative bilirubin level was 23 μmol/L. A cutoff preoperative bilirubin level of 75 μmol/L was derived from Youden’s index (sensitivity 0.333; specificity 0.949) and confirmed to be optimal by logistic regression (relative risk 9.250; 95% confidence interval 1.932–44.291; *p* = 0.005), with mortality shown to be statistically different at 90 days (*p* = 0.008). Patients were divided into Group A (≤75 μmol/L; *n* = 82) and Group B (> 75 μmol/L; n = 8). Group B had a higher preoperative bilirubin level (*p* < 0.001), more intraoperative blood loss (3.12 vs 1.4 L; *p* = 0.008), transfusion (100% vs 42.0%; *p* = 0.011) and replacement (2.45 vs 0.0 L; *p* < 0.001), more postoperative renal complications (*p* = 0.036), more in-hospital deaths (50% vs 8.5%; *p* = 0.004), and more 90-day deaths (50% vs 9.8%; *p* = 0.008). Group A had a longer follow-up period (p = 0.008). The groups were otherwise comparable. Disease-free survival was similar between groups (*p* = 0.142) but overall survival was better in Group A (5-year, 25.2% vs 0%; *p* < 0.001). On multivariate analysis, preoperative bilirubin level and intraoperative blood replacement were risk factors for 90-day mortality.

**Conclusion:**

A cutoff value of preoperative bilirubin level of 75 μmol/L is suggested, as the study showed that a preoperative bilirubin level ≤ 75 μmol/L resulted in significantly less blood replacement necessitated by blood loss during operation and significantly better patient survival after surgery.

## Background

Hilar cholangiocarcinoma (HC) refers to tumors developing in the right or left hepatic duct or both ducts at or near the biliary confluence. It is a rare cancer but is the most common type of biliary cancer and accounts for up to 70% of all primary tumors of the biliary tract [1–5]. It was also called Klatskin tumor after description of the condition by Dr. Gerald Klatskin in 1965 [6]. Complete resection is regarded as the most effective therapy for HC [7–9] but it may entail high postoperative mortality as a result of liver failure and/or infectious complications [10]. As most of the patients initially present with jaundice, preoperative biliary drainage – first reported by Nakayama in 1978 – may reduce the risks associated with preoperative jaundice [11]. Various options including endoscopic retrograde cholangiopancreatography (ERCP), endoscopic nasobiliary drainage (ENBD) and percutaneous transhepatic biliary drainage (PTBD) have been advocated as modalities for biliary drainage. However, the optimal preoperative level of bilirubin has not been determined. A preoperative bilirubin level of < 3 mg/dL (51 μmol/L) was recommended by Makuuchi et al. [12] and Nimura et al. [13], as a level of > 3 mg/dL (51 μmol/L) had been found to be a negative factor affecting overall survival [7]. A preoperative bilirubin level of > 10 mg/dL (171 μmol/L) was significantly associated with postoperative mortality [14], while a level of 4.4 mg/dL (75 μmol/L) could reduce postoperative morbidity [15]. The aim of this retrospective study was to identify the optimal level of bilirubin before hepatectomy with curative intent for HC in relation to postoperative complication and mortality.

## Methods

This study has been approved by the Institutional Review Board of the University of Hong Kong/Hospital Authority Hong Kong West Cluster (IRB Reference Number: UW 20–578). All patients gave their written informed consent to collection and use of their data for research purposes. No individual patients can be identified by the anonymous data used in this study.

Clinical data of HC patients at a single center in the period from January 1989 to December 2014 were reviewed. The study included patients who received hepatectomy with curative intent for HC and excluded patients found to have unresectable HC during initial investigation or during operation. Postoperative complications were graded according to the Clavien-Dindo descriptions [16]. Major complication (grade IIIA or above) and 90-day mortality were the endpoints.

To evaluate the extent of biliary obstruction and to relieve obstructive jaundice before hepatectomy, the patients underwent preoperative biliary anatomical investigation and drainage in the form of PTBD or ERCP with internal stenting or both. Types of stricture were decided by the location and extent of biliary involvement and were classified according to the Bismuth-Corlette classification [17]. PTBD was performed primarily on the side of the future liver remnant. An 8-Fr drainage catheter with guidewire was placed with its tip in the bile duct, draining externally to a bag. ERCP was performed with insertion of larger-bore straight or double pigtail stent. Stent exchange was performed only if acute cholangitis recurred. PTBD would be performed if ERCP failed to bring down a patient’s bilirubin level, and vice versa. ENBD was not routinely performed. For major right-sided hepatic resection with a future liver remnant (the left lobe) estimated to be < 30% of the estimated standard liver volume [18, 19], preoperative portal vein embolization was needed; 4 to 6 weeks after the portal vein embolization, contrast computed tomographic scan of the abdomen with volumetric measurement was performed to assess the future liver remnant size and operability.

During surgical resection, left or right hepatectomy with or without caudate lobectomy was carried out. In radical lymphadenectomy, regional and celiac lymph nodes were dissected. In extended radical lymphadenectomy, para-aortic lymph nodes were also dissected. Only some patients received extended radical lymphadenectomy. In the later period of the study, intraoperative confirmation of negative resection margin was routinely performed. Parenchymal transection was performed along the demarcation line with an ultrasonic dissector (Cavitron Ultrasonic Surgical Aspirator; Valleylab, Boulder, CO, USA) with or without portal inflow clamping. Bilioenteric continuity was re-established by Roux-en-Y hepaticojejunostomy. The jejunal limb was brought to the hepatic ducts via the retrocolic-retrogastric route. The PTBD catheter on the side of the liver remnant was kept for about 4 weeks and spigotted at around postoperative day 5.

In the later period of the study, some patients received adjuvant chemotherapy or radiotherapy. A standard dose was used as long as it was tolerated. There was no established use of chemotherapy agents, but gemcitabine, cisplatin and fluorouracil were commonly used.

All cancer staging followed the Tumor-Node-Metastasis staging (7th edition) by the Union for International Cancer Control [20]. In-hospital mortality was defined as death happening during the hospital stay for hepatic resection. All patients had postoperative follow-up by same team of surgeons every 1 to 3 months in the first year. Liver biochemistry was monitored at each follow-up visit, and computed tomographic scan of the abdomen was performed every 3 to 6 months in the first year and every 6 months thereafter for monitoring for recurrent disease. None of the patients was lost to follow-up during the study period.

### Statistical analysis

Continuous variables were described as medians with range in brackets. The Mann-Whitney U test or the t-test, where appropriate, was used to analyze parametric variables. Pearson’s chi-squared test or Fisher’s exact test, where appropriate, was used to analyze categorical variables. Youden’s index was used to estimate the optimal preoperative level of bilirubin. The receiver operating characteristic curve was used to test the sensitivity and specificity of the value, and logistic regression was used to show how the bilirubin level would affect 90-day mortality. The Kaplan-Meier method was used to calculate survival. Overall survival was calculated from the day after operation to the day of last follow-up visit or death. Disease-free survival was calculated from the day of discharge to the day of disease recurrence. The log-rank test was used for comparison of survival between groups. Univariate and multivariate analyses by logistic regression were performed to look for risk factors for 90-day mortality. *P* values < 0.05 denoted statistical significance. The computer software Statistical Product and Service Solutions, version 20 (IBM Corporation, Armonk, NY, USA), was used for all statistical analyses.

## Results

In the 308 patients reviewed, 200 patients were excluded from analysis due to advanced disease or metastasis found on preoperative imaging and 18 patients were excluded due to inoperable disease confirmed intraoperatively. Ninety patients (63 male and 27 female; median age, 66 years) who underwent curative treatment for HC were included in the study. Almost half of them (45.6%) had medical comorbidities. Their median preoperative bilirubin level was 23 μmol/L. Eight-six patients (95.6%) underwent preoperative biliary drainage: 32 (32/86 = 37.2%) had ERCP alone, 10 (10/86 = 11.6%) had PTBD alone, and 45 (45/86 = 52.3%) had both. All patients received major liver resection, with a median operation time of 690 min. The median intraoperative blood loss was 1.5 L, and 41 patients (46.6%) required blood transfusion. Postoperative complications occurred in 52 patients, while 32 of them (35.6%) had complications of Clavien-Dindo grade IIIA or above. The in-hospital mortality rate was 12.2% (*n* = 11): 3 patients died of liver failure and 8 patients died of sepsis related to multi-organ failure. The median hospital stay was 20.5 days. On histopathological examination, 42 patients had a positive resection margin whereas 48 patients had a clear margin. Microvascular invasion was present in 28 patients (31.1%). Fifteen patients (16.7%) received adjuvant radiotherapy and/or chemotherapy in the later part of the study period. The median follow-up time was 21.1 months, and the median time to disease recurrence was 11.8 months. The median disease-free survival was 16.8 months and the median overall survival was 22 months. The 5-year overall survival rate was 22.9%.

The 90 patients were divided into two groups according to their bilirubin level before hepatectomy: Group A with bilirubin ≤75 μmol/L (*n* = 82) and Group B with bilirubin > 75 μmol/L (n = 8). The cutoff value of 75 μmol/L (4.4 mg/dL) was derived from Youden’s index, with a sensitivity of 0.333 and specificity of 0.949. This level was confirmed to be optimal with a relative risk of 9.250 (95% confidence interval 1.932–44.291; *p* = 0.005) in logistic regression, and the mortality was shown to be statistically different at 90 days (*p* = 0.008) (Table 1).

**Table 1:** Preoperative bilirubin level of 75 μmol/L on 90-day mortality

	≤75 μmol/L (*n* = 82)	> 75 μmol/L (*n* = 8)	*P* value
90-day mortality			0.008^a^
No	74 (90.2%)	4 (50%)	
Yes	8 (9.8%)	4 (50%)	

^a^statistically significant

Table 2 is a comparison of the two groups of patients in terms of demographic and preoperative characteristics. They were comparable in all the demographic and preoperative parameters except post-drainage bilirubin level (*p* < 0.001). Drainage brought down the median bilirubin level from 98.0 μmol/L (range, 8–576 μmol/L) to 20.5 μmol/L (range, 7–73 μmol/L) in Group A and from 124.0 μmol/L (range, 28–492 μmol/L) to 95.5 μmol/L (range, 76–366 μmol/L) in Group B. The median time from drainage procedure to hepatectomy was 1.84 months in Group A and 1.35 months in Group B.

**Table 2:** Comparison of the two groups of patients in terms of demographic and preoperative characteristics

	Bilirubin ≤75 μmol/L (*n* = 82)	Bilirubin > 75 μmol/L (*n* = 8)	*P* value
Age (years)	66.0 (29–85)	69.0 (56–72)	0.696
Male: Female	55: 27	8: 0	0.125
Comorbidity	38 (46.3%)	3 (37.5%)	0.914
Heart	35 (42.7%)	2 (25%)	0.553
Lung	5 (6.1%)	1 (12.5%)	1.000
Renal	1 (1.2%)	0 (0%)	1.000
Diabetes mellitus	15 (18.3%)	1 (12.5%)	1.000
Child-Pugh class			0.159
A	37 (45.1%)	1 (12.5%)	
B	45 (54.9%)	7 (87.5%)	
Pre-drainage bilirubin (μmol/L)	98.0 (8–576)	124.0 (28–492)	0.673
Post-drainage bilirubin (μmol/L)	20.5 (7–73)	95.5 (76–366)	< 0.001^a^
Creatinine (μmol/L)	76.5 (40–148)	81.0 (61–130)	0.391
Albumin (g/L)	37.0 (27–51)	37.0 (26–43)	0.312
International normalized ratio	1.0 (0.9–1.4)	1.0 (0.9–1.2)	0.473
Platelet count (× 10^9^/L)	288.0 (71–561)	342.0 (166–699)	0.750
Carcinoembryonic antigen (ng/mL)	2.65 (0.3–16)	3.0 (1.8–10.0)	0.528
Indocyanine green retention rate at 15 min (%) (*n* = 38)	10.55 (3–20.1)	20.6 (7.9–32.5)	0.148
Aspartate transaminase (U/L)	71.5 (14–748)	56.5 (45–337)	0.771
Alanine transaminase (U/L)	101.5 (9–1178)	106.0 (37–481)	0.837
Neoadjuvant chemotherapy	1 (1.6%)	0 (0%)	1.000
Portal vein embolization	28 (34.1%)	2 (25.0%)	0.896
Time from drainage procedure to hepatectomy (months)	1.84 (0.2–4.7)	1.35 (0.3–3.1)	0.772
ERCP	69 (84.1%)	7 (87.5%)	1.000
ERCP purpose			0.939
No	13 (15.9%)	1 (12.5%)	
Diagnosis	53 (64.6%)	5 (62.5%)	
Complication	1 (1.2%)	0 (0%)	
Diagnosis + Complication	15 (18.3%)	2 (25%)	
ERCP complication			0.615
No	57 (69.5%)	6 (75%)	
Cholangitis	10 (12.2%)	2 (25%)	
Pancreatitis	11 (13.4%)	0 (0%)	
Deranged liver function tests	2 (2.4%)	0 (0%)	
Slip	1 (1.2%)	0 (0%)	
Cholangitis + Pancreatitis	1 (1.2%)	0 (0%)	
PTBD	49 (59.8%)	5 (62.5%)	1.000
PTBD purpose			0.755
No	34 (41.5%)	3 (37.5%)	
Diagnosis	30 (36.6%)	4 (50%)	
Complication	4 (4.9%)	0 (0%)	
Diagnosis + Complication	14 (17.1%)	1 (12.5%)	
PTBD complication			0.722
No	66 (80.5%)	7 (87.5%)	
Cholangitis	4 (4.9%)	1 (12.5%)	
Cholecystitis	1 (1.2%)	0 (0%)	
Severe pancreatitis precluding management	1 (1.2%)	0 (0%)	
Slip	6 (7.3%)	0 (0%)	
Cholangitis + Slip	4 (4.9%)	0 (0%)	

Data are shown as number of patient (percent) or median (range) or ratio

^a^statistically significant

Table 3 compares the two groups of patients in terms of intraoperative, postoperative and histopathological results. Although the two groups were comparable in terms of operation time (*p* = 0.156), resection type (*p* = 0.558), and major vascular resection and reconstruction (*p* = 0.210), Group B had significantly more intraoperative blood loss (median, 3.12 L vs 1.4 L; *p* = 0.008), blood transfusion (100% vs 42.0%; *p* = 0.011), and blood replacement (median, 2.45 L vs 0.0 L; *p* < 0.001). Significantly more patients in Group B had renal complications (37.5% vs 7.3%; *p* = 0.036). This group also had significantly more in-hospital deaths (grade-V complication) (50% vs 8.5%; *p* = 0.004) and 90-day deaths (50% vs 9.8%; *p* = 0.008). The follow-up duration was significantly longer in Group A (median, 21.6 months vs 4.1 months; p = 0.008). The two groups were otherwise comparable in postoperative details. On histopathological examination, they also had similar results.

**Table 3:** Comparison of the two groups of patients in terms of intraoperative, postoperative and histopathological results

	Bilirubin ≤75 μmol/L (*n* = 82)	Bilirubin > 75 μmol/L (*n* = 8)	*P* value
Blood loss (L)	1.4 (0.3–20)	3.12 (1.4–6.0)	0.008^a^
Blood replacement (L)	0.0 (0.0–13.8)	2.45 (0.6–4.3)	< 0.001^a^
Blood transfusion	34 (42.0%)	8 (100%)	0.011^a^
Operation time (min)	674.0 (222–1290)	797.0 (514–960)	0.156
Type of resection			0.558
Right hepatectomy	5 (6.1%)	1 (12.5%)	
Right extended hepatectomy	3 (3.7%)	0 (0%)	
Left hepatectomy	2 (2.4%)	1 (12.5%)	
Left extended hepatectomy	2 (2.4%)	0 (0%)	
Right trisectionectomy	2 (2.4%)	0 (0%)	
Left trisectionectomy	1 (1.2%)	0 (0%)	
Right hepatectomy + caudate	26 (31.7%)	1 (12.5%)	
Right extended hepatectomy + caudate	7 (8.5%)	0 (0%)	
Left hepatectomy + caudate	12 (14.6%)	3 (37.5%)	
Left extended hepatectomy + caudate	5 (6.1%)	0 (0%)	
Right trisectionectomy + caudate	17 (20.7%)	2 (25%)	
Blood vessel reconstruction	22 (26.8%)	0 (0%)	0.210
Portal vein reconstruction	17 (20.7%)	0 (0%)	0.339
Hepatic artery reconstruction	5 (6.1%)	0 (0%)	1.000
Patients with postoperative complications	46 (56.1%)	6 (75%)	0.510
Renal complications	6 (7.3%)	3 (37.5%)	0.036^a^
Urinary tract infection	1 (1.2%)	1 (12.5%)	0.418
Renal failure	5 (6.1%)	2 (25%)	0.225
Patients with complications of grade IIIA or above	28 (34.1%)	4 (50%)	0.612
Grade IIIA	24 (29.3%)	3 (37.5%)	0.936
Grade IIIB	14 (17.1%)	1 (12.5%)	1.000
Grade IVA	6 (7.3%)	1 (12.5%)	1.000
Grade IVB	0 (0%)	0 (0%)	///
Grade V	7 (8.5%)	4 (50%)	0.004^a^
In-hospital mortality	7 (8.5%)	4 (50%)	0.004^a^
30-day mortality	5 (6.1%)	2 (25%)	0.225
90-day mortality	8 (9.8%)	4 (50%)	0.008^a^
Hospital stay (days)	20.0 (6–93)	27.5 (10–214)	0.228
Pattern of recurrence			0.394
No recurrence	31 (37.8%)	5 (62.5%)	
Intrahepatic recurrence	7 (8.5%)	0 (0%)	
Extrahepatic recurrence	22 (26.8%)	2 (25%)	
Intrahepatic and extrahepatic recurrence	22 (26.8%)	1 (12.5%)	
Time to recurrence (months)	16.7 (1.2–218.3)	11.6 (1.1–24.4)	0.311
Follow-up duration (months)	21.6 (0.4–218.3)	4.1 (0.3–26.6)	0.008^a^
Resection margin			1.000
Not involved	44 (53.7%)	4 (50%)	
Involved	38 (46.3%)	4 (50%)	
Invasion of major branch of portal or hepatic vein			1.000
No	70 (85.4%)	7 (87.5%)	
Yes	12 (14.6%)	1 (12.5%)	
Microvascular invasion			0.429
Absent	55 (67.1%)	7 (87.5%)	
Present	27 (32.9%)	1 (12.5%)	
Number of lymph node metastasis	0.0 (0–25)	0.0 (0–3)	0.891
Klatskin staging			0.208
I	12 (15.0%)	0 (0%)	
II	25 (31.3%)	3 (37.5%)	
IIIA	2 (2.5%)	1 (12.5%)	
IIIB	26 (32.5%)	4 (50%)	
IVA	12 (15.0%)	0 (0%)	
IVB	3 (3.8%)	0 (0%)	

Data are shown as number of patient (percent) or median (range)

^a^statistically significant

**Fig. 1:**
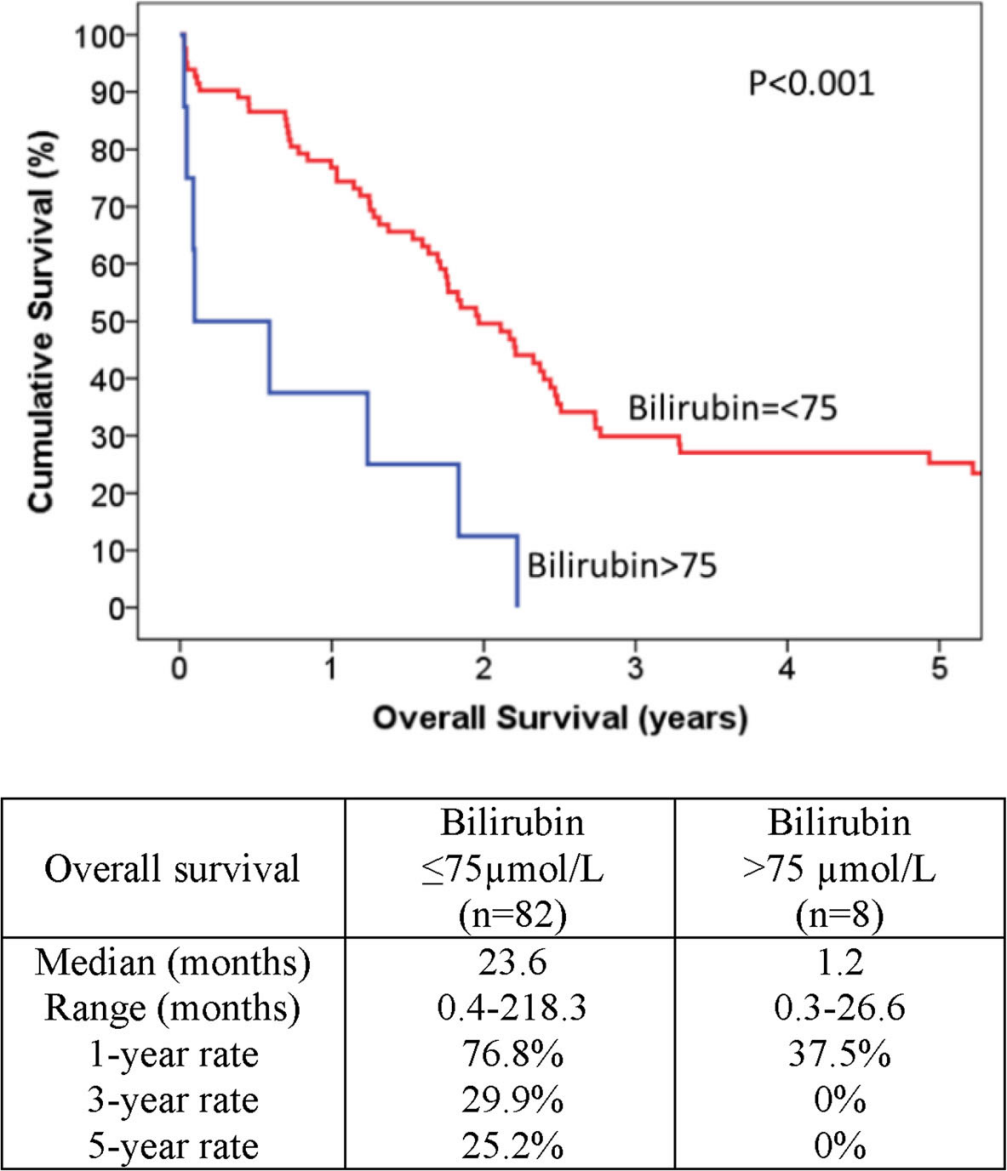
Comparison of postoperative survival in the two groups of patients

Disease-free survival did not differ much between the two groups (*p* = 0.142). The median disease-free survival was 16.8 months in Group A and 7.1 months in Group B. The 5-year disease-free survival rate was 24.8% in Group A and 0% in Group B. However, as detailed in Fig. 1, overall survival was significantly better in Group A (*p* < 0.001).

Since the two groups were comparable in overall and major complications and significant differences were found in grade-V complication and 90-day mortality, univariate and multivariate analyses by logistic regression were performed to identify risk factors for 90-day mortality only. As shown in Table 4, bilirubin group (≤ 75 μmol/L vs > 75 μmol/L) before surgery and blood replacement resulting from blood loss in operation were the two factors found to predict 90-day mortality.

**Table 4:** Univariate and multivariate analyses of factors for 90-day mortality

	Univariate analysis			Multivariate analysis		
	Exp(B)	95% CI	*P* value	Exp(B)	95% CI	*P* value
Blood loss	1.593	1.109–2.289	0.012^a^	–	–	–
Intraoperative blood replacement	1.915	1.195–3.07	0.007^a^	1.71	1.056–2.768	0.029^a^
Preoperative bilirubin	1.005	0.996–1.015	0.24	–	–	–
Bilirubin group (≤75 vs > 75 μmol/L)	9.25	1.932–44.291	0.005^a^	7.048	1.129–44.002	0.037^a^

^a^statistically significant

## Discussion

Clinical manifestation of HC depends on stage and location of the tumor [4, 21]. Most patients present with obstructive jaundice [21]. Occasionally abdominal pain or acute cholangitis is also present. The aims of HC management are to deal with acute complications such as cholangitis, confirm the diagnosis, assess the general condition, liver function and liver remnant after resection, and check the anatomy and extent of the tumor in relation to the hepatic artery and portal vein in order to determine resectability. Liver function is in general impaired in HC patients. Hyperbilirubinemia is common as a result of obstructive jaundice. Unfortunately, it causes cholestasis and increases the risk of biliary infection and impairs cellular immunity [22]. Furthermore, liver resection in jaundiced patients carries a high risk of postoperative mortality and morbidity [23]. Therefore, various options of biliary decompression before hepatectomy have been advocated.

Intraoperative blood loss is associated with poor survival in several types of cancer, including gastric cancer [24, 25] and pancreatic cancer [26]. At the same time, blood transfusion is known to be an adverse factor in cancer surgery. It causes dysfunction of the immune system and may adversely affect the outcomes of cancer patients [27–29]. Moreover, blood transfusion has been identified as the single independent prognostic factor for post-hepatectomy liver failure [30]. In the present study, Group A and Group B were similar in platelet count, international normalized ratio and resection extent but Group B was significantly poor when it comes to intraoperative blood loss, blood transfusion and blood replacement. This could have been due to hyperbilirubinemia affecting coagulopathy. The optimal level of bilirubin remains unestablished, but overall speaking, the higher the level, the greater the chance of postoperative morbidity and mortality. As such, preoperative drainage of the biliary system is advocated. In the present cohort, postoperative mortality was significantly worse in patients with a preoperative bilirubin level > 75 μmol/L.

Biliary decompression may relieve jaundice (which in turn improves coagulopathy), improve renal insufficiency associated with liver failure, and provide symptomatic relief [31]. Drainage is therefore advocated, with the aim of reducing risks of cholangitis and postoperative liver failure [32, 33]. The use and route of preoperative biliary drainage in jaundiced patients remain controversial [34–36]. There is no definite cutoff value of bilirubin for major hepatectomy for HC. Various bilirubin cutoff levels have been recommended, and it has been suggested that operation can be performed when the bilirubin level is brought down to < 2–3 mg/dL (34–51 μmol/L) by preoperative biliary decompression [7, 12, 13, 37]. Jaundice has been shown to affect morbidity and decrease survival in pancreaticoduodenectomy, and drainage with stenting has been suggested for HC management [38]. Although post-procedural cholangitis – one of the serious complications of preoperative biliary drainage – has been reported to be one of the most important predicative factors for liver failure and mortality after major hepatectomy [39, 40], biliary drainage is generally indicated in septic patients having cholangitis, portal vein embolization, or chemotherapy.

This study has its own limitations. Firstly, the series is small (90 patients in total), especially the hyperbilirubinemic cohort (8 patients). We admit that a cohort of 8 patients only is not a powerful support for the optimal bilirubin level identified. However, it is a matter of fact that HC was an uncommon disease and most patients presented late and thus were inoperable, which further reduced the number of patients having hepatectomy with curative intent for the disease. Secondly, this was a single-center study, which had inherent limitations in inter-observer variability and treatment heterogeneity in perioperative management and operative technique. On the other hand, because of the study’s retrospective nature, missing data, possibility of selection bias and treatment heterogeneity throughout the years could not be completely avoided. While it would be most ideal to perform a meta-analysis to analyze reported results on this topic and to identify the level at risk for morbidity and mortality, this retrospective analysis with a reasonable sample size still serves to shed some light on HC management.

## Conclusions

In the management of operable HC patients with hyperbilirubinemia, preoperative biliary drainage should be performed to bring down the bilirubin to a certain level in order to reduce postoperative complications and mortality. This study identified that a cutoff level of 75 μmol/L was one of the two prognostic factors for 90-day mortality and demonstrated that a preoperative bilirubin level ≤ 75 μmol/L resulted in significantly less blood replacement necessitated by blood loss during operation and significantly better patient survival after surgery.

## Data Availability

The datasets used and analyzed during the current study are available from the corresponding author on reasonable request.

## Abbreviations

ENBD: Endoscopic nasobiliary drainage
ERCP: Endoscopic retrograde cholangiopancreatography
HC: Hilar cholangiocarcinoma
PTBD: Percutaneous transhepatic biliary drainage

## References

[1] Burke EC, Jarnagin WR, Hochwald SN, Pisters PW, Fong Y, Blumgart LH (1998). Hilar Cholangiocarcinoma: patterns of spread, the importance of hepatic resection for curative operation, and a presurgical clinical staging system. Ann Surg.

[2] DeOliveira ML, Cunningham SC, Cameron JL, Kamangar F, Winter JM, Lillemoe KD, Choti MA, Yeo CJ, Schulick RD (2007). Cholangiocarcinoma: thirty-one-year experience with 564 patients at a single institution. Ann Surg.

[3] Launois B, Reding R, Lebeau G, Buard JL (2000). Surgery for hilar cholangiocarcinoma: French experience in a collective survey of 552 extrahepatic bile duct cancers. J Hepatobiliary Pancreat Surg.

[4] Nakeeb A, Pitt HA, Sohn TA, Coleman J, Abrams RA, Piantadosi S, Hruban RH, Lillemoe KD, Yeo CJ, Cameron JL (1996). Cholangiocarcinoma. A spectrum of intrahepatic, perihilar, and distal tumors. Ann Surg.

[5] Welzel TM, McGlynn KA, Hsing AW, O'Brien TR, Pfeiffer RM (2006). Impact of classification of hilar cholangiocarcinomas (Klatskin tumors) on the incidence of intra- and extrahepatic cholangiocarcinoma in the United States. J Natl Cancer Inst.

[6] Klatskin G (1965). Adenocarcinoma of the Hepatic Duct at Its Bifurcation within the Porta Hepatis. An Unusual Tumor with Distinctive Clinical and Pathological Features. Am J Med.

[7] Cho MS, Kim SH, Park SW, Lim JH, Choi GH, Park JS, Chung JB, Kim KS (2012). Surgical outcomes and predicting factors of curative resection in patients with hilar cholangiocarcinoma: 10-year single-institution experience. J Gastrointest Surg.

[8] Ito F, Agni R, Rettammel RJ, Been MJ, Cho CS, Mahvi DM, Rikkers LF, Weber SM (2008). Resection of hilar cholangiocarcinoma: concomitant liver resection decreases hepatic recurrence. Ann Surg.

[9] Launois B, Campion JP, Brissot P, Gosselin M (1979). Carcinoma of the hepatic hilus. Surgical management and the case for resection. Ann Surg.

[10] Jarnagin WR, Fong Y, DeMatteo RP, Gonen M, Burke EC, Bodniewicz BJ, Youssef BM, Klimstra D, Blumgart LH (2001). Staging, resectability, and outcome in 225 patients with hilar cholangiocarcinoma. Ann Surg.

[11] Nakayama T, Ikeda A, Okuda K (1978). Percutaneous transhepatic drainage of the biliary tract: technique and results in 104 cases. Gastroenterology.

[12] Makuuchi M, Thai BL, Takayasu K, Takayama T, Kosuge T, Gunven P, Yamazaki S, Hasegawa H, Ozaki H (1990). Preoperative portal embolization to increase safety of major hepatectomy for hilar bile duct carcinoma: a preliminary report. Surgery.

[13] Nimura Y, Hayakawa N, Kamiya J, Kondo S, Shionoya S (1990). Hepatic segmentectomy with caudate lobe resection for bile duct carcinoma of the hepatic hilus. World J Surg.

[14] Su CH, Tsay SH, Wu CC, Shyr YM, King KL, Lee CH, Lui WY, Liu TJ, P'Eng FK (1996). Factors influencing postoperative morbidity, mortality, and survival after resection for hilar cholangiocarcinoma. Ann Surg.

[15] Grandadam S, Compagnon P, Arnaud A, Olivie D, Malledant Y, Meunier B, Launois B, Boudjema K (2010). Role of preoperative optimization of the liver for resection in patients with hilar cholangiocarcinoma type III. Ann Surg Oncol.

[16] Dindo D, Demartines N, Clavien PA (2004). Classification of surgical complications: a new proposal with evaluation in a cohort of 6336 patients and results of a survey. Ann Surg.

[17] Bismuth H, Nakache R, Diamond T (1992). Management strategies in resection for hilar cholangiocarcinoma. Ann Surg.

[18] Chan SC, Lo CM, Chok KS, Sharr WW, Cheung TT, Tsang SH, Chan AC, Fan ST (2011). Validation of graft and standard liver size predictions in right liver living donor liver transplantation. Hepatol Int.

[19] Urata K, Kawasaki S, Matsunami H, Hashikura Y, Ikegami T, Ishizone S, Momose Y, Komiyama A, Makuuchi M (1995). Calculation of child and adult standard liver volume for liver transplantation. Hepatology.

[20] Liver ES (2009). AJCC cancer staging manual.

[21] Blechacz B, Komuta M, Roskams T, Gores GJ (2011). Clinical diagnosis and staging of cholangiocarcinoma. Nat Rev Gastroenterol Hepatol.

[22] van der Gaag NA, Kloek JJ, de Castro SM, Busch OR, van Gulik TM, Gouma DJ (2009). Preoperative biliary drainage in patients with obstructive jaundice: history and current status. J Gastrointest Surg.

[23] Belghiti J, Hiramatsu K, Benoist S, Massault P, Sauvanet A, Farges O (2000). Seven hundred forty-seven hepatectomies in the 1990s: an update to evaluate the actual risk of liver resection. J Am Coll Surg.

[24] Ito Y, Kanda M, Ito S, Mochizuki Y, Teramoto H, Ishigure K, Murai T, Asada T, Ishiyama A, Matsushita H, et al. Intraoperative Blood Loss is Associated with Shortened Postoperative Survival of Patients with Stage II/III Gastric Cancer: Analysis of a Multi-institutional Dataset. World J Surg. 2019;43(3):870–7. 10.1007/s00268-018-4834-0 30377722

[25] Kamei T, Kitayama J, Yamashita H, Nagawa H (2009). Intraoperative blood loss is a critical risk factor for peritoneal recurrence after curative resection of advanced gastric cancer. World J Surg.

[26] Nagai S, Fujii T, Kodera Y, Kanda M, Sahin TT, Kanzaki A, Yamada S, Sugimoto H, Nomoto S, Takeda S (2011). Impact of operative blood loss on survival in invasive ductal adenocarcinoma of the pancreas. Pancreas.

[27] Baumgartner JM, Silliman CC, Moore EE, Banerjee A, McCarter MD (2009). Stored red blood cell transfusion induces regulatory T cells. J Am Coll Surg.

[28] Yamashita K, Sakuramoto S, Kikuchi S, Katada N, Kobayashi N, Watanabe M (2007). Transfusion alert for patients with curable cancer. World J Surg.

[29] Zhou HY, Yi W, Wang J, Zhang J, Wang WJ, Hu ZQ (2014). Association of perioperative allogeneic blood transfusions and prognosis of patients with gastric cancer after curative gastrectomy. Am J Surg.

[30] Dumitrascu T, Brasoveanu V, Stroescu C, Ionescu M, Popescu I (2016). Major hepatectomies for perihilar cholangiocarcinoma: Predictors for clinically relevant postoperative complications using the International Study Group of Liver Surgery definitions. Asian J Surg.

[31] Sarmiento JM, Nagorney DM (2002). Hepatic resection in the treatment of perihilar cholangiocarcinoma. Surg Oncol Clin N Am.

[32] Liu F, Li Y, Wei Y, Li B (2011). Preoperative biliary drainage before resection for hilar cholangiocarcinoma: whether or not? A systematic review. Dig Dis Sci.

[33] Tsai HM, Chuang CH, Lin XZ, Chen CY (2009). Factors relating to the short term effectiveness of percutaneous biliary drainage for hilar cholangiocarcinoma. World J Gastroenterol.

[34] Al Mahjoub A, Menahem B, Fohlen A, Dupont B, Alves A, Launoy G, Lubrano J (2017). Preoperative Biliary Drainage in Patients with Resectable Perihilar Cholangiocarcinoma: Is Percutaneous Transhepatic Biliary Drainage Safer and More Effective than Endoscopic Biliary Drainage? A Meta-Analysis. J Vasc Interv Radiol.

[35] Celotti A, Solaini L, Montori G, Coccolini F, Tognali D, Baiocchi G (2017). Preoperative biliary drainage in hilar cholangiocarcinoma: Systematic review and meta-analysis. Eur J Surg Oncol.

[36] Liu JG, Wu J, Wang J, Shu GM, Wang YJ, Lou C, Zhang J, Du Z (2018). Endoscopic Biliary Drainage Versus Percutaneous Transhepatic Biliary Drainage in Patients with Resectable Hilar Cholangiocarcinoma: A Systematic Review and Meta-Analysis. J Laparoendosc Adv Surg Tech A.

[37] Mansour JC, Aloia TA, Crane CH, Heimbach JK, Nagino M, Vauthey JN (2015). Hilar cholangiocarcinoma: expert consensus statement. HPB.

[38] Sauvanet A, Boher JM, Paye F, Bachellier P, Sa Cuhna A, Le Treut YP, Adham M, Mabrut JY, Chiche L, Delpero JR (2015). Severe Jaundice Increases Early Severe Morbidity and Decreases Long-Term Survival after Pancreaticoduodenectomy for Pancreatic Adenocarcinoma. J Am Coll Surg.

[39] Ribero D, Zimmitti G, Aloia TA, Shindoh J, Fabio F, Amisano M, Passot G, Ferrero A, Vauthey JN (2016). Preoperative Cholangitis and Future Liver Remnant Volume Determine the Risk of Liver Failure in Patients Undergoing Resection for Hilar Cholangiocarcinoma. J Am Coll Surg.

[40] Wiggers JK, Groot Koerkamp B, Cieslak KP, Doussot A, van Klaveren D, Allen PJ, Besselink MG, Busch OR, D'Angelica MI, DeMatteo RP (2016). Postoperative Mortality after Liver Resection for Perihilar Cholangiocarcinoma: Development of a Risk Score and Importance of Biliary Drainage of the Future Liver Remnant. J Am Coll Surg.

